# Supplementary material for: Protective role of brain derived neurotrophic factor (BDNF) in obstructive sleep apnea syndrome (OSAS) patients
Source: PLoS One. 2020 Jan 17;15(1):e0227834. doi: 10.1371/journal.pone.0227834 (PMC6968866; doi:10.1371/journal.pone.0227834)
Supplement: Attachment — Submitted filename: Response To Reviewers.docx [file pone.0227834.s002.docx]

REBUTTAL LETTER

Dear Editor

We have greatly appreciated the referees’ comments and suggestions. Their constructive criticisms, allowed us to improve the quality and the significance of the results of the submitted manuscript.

Our revision was detailed below.

The manuscript respects the PLOS ONE’s style requirements.

A carefully review of the manuscript by an English mother tongue has been done. Actually, the manuscript is technically sound and the data does support the conclusions.

The statistical analysis was appropriately and rigorously reviewed.

We make all data underlying the findings fully available.

We added additional patients’ cohort details including respiratory function findings, blood gas analysis and presence of comorbidities. Therefore, a new Table 1 was added to the study.

I am looking forward to hearing from you and I would like to thank you in advance for your attention and your time.

With my best personal regards.

Alberto Ricci MD
